# Modeling the Potential Future Distribution of Anthrax Outbreaks under Multiple Climate Change Scenarios for Kenya

**DOI:** 10.3390/ijerph18084176

**Published:** 2021-04-15

**Authors:** Fredrick Tom Otieno, John Gachohi, Peter Gikuma-Njuru, Patrick Kariuki, Harry Oyas, Samuel A. Canfield, Bernard Bett, Moses Kariuki Njenga, Jason K. Blackburn

**Affiliations:** 1 Animal Health Program, International Livestock Research Institute, P.O. Box 30709 Nairobi 00100, Kenya; b.bett@cgiar.org; 2 School of Environment, Water and Natural Resources, South Eastern Kenya University, P.O. Box 17, Kitui 90200, Kenya; pnjuru@seku.ac.ke (P.G.-N.); pkariuki@seku.ac.ke (P.K.); 3 Paul Allen School for Global Health, Washington State University-Global Health Kenya, One Padmore Place, George Padmore Lane, P.O. Box 19676 Nairobi 00100, Kenya; john.gachohi@wsu.edu (J.G.); mkariuki.njenga@wsu.edu (M.K.N.); 4 School of Public Health, Jomo Kenyatta University of Agriculture and Technology, P.O. Box 62000, Nairobi 00200, Kenya; 5 Veterinary Epidemiology and Economics Unit, Kenya Ministry of Agriculture, Livestock and Fisheries, P.O. Box 30028 Nairobi 00100, Kenya; harryoyas@gmail.com; 6 Spatial Epidemiology and Ecology Research Laboratory, Department of Geography, University of Florida, Gainesville, FL 32611, USA; scanfield@ufl.edu (S.A.C.); jkblackburn@ufl.edu (J.K.B.); 7 Emerging Pathogens Institute, University of Florida, 2055 Mowry Road, Gainesville, FL 32611, USA

**Keywords:** anthrax, risk, livestock, spatial, geographic, distribution, climate, change, ecological, modelling, Kenya

## Abstract

The climate is changing, and such changes are projected to cause global increase in the prevalence and geographic ranges of infectious diseases such as anthrax. There is limited knowledge in the tropics with regards to expected impacts of climate change on anthrax outbreaks. We determined the future distribution of anthrax in Kenya with representative concentration pathways (RCP) 4.5 and 8.5 for year 2055. Ecological niche modelling (ENM) of boosted regression trees (BRT) was applied in predicting the potential geographic distribution of anthrax for current and future climatic conditions. The models were fitted with presence-only anthrax occurrences (n = 178) from historical archives (2011–2017), sporadic outbreak surveys (2017–2018), and active surveillance (2019–2020). The selected environmental variables in order of importance included rainfall of wettest month, mean precipitation (February, October, December, July), annual temperature range, temperature seasonality, length of longest dry season, potential evapotranspiration and slope. We found a general anthrax risk areal expansion i.e., current, 36,131 km^2^, RCP 4.5, 40,012 km^2^, and RCP 8.5, 39,835 km^2^. The distribution exhibited a northward shift from current to future. This prediction of the potential anthrax distribution under changing climates can inform anticipatory measures to mitigate future anthrax risk.

## 1. Introduction

Anthrax is an important zoonotic disease caused by a soil-borne, spore-forming bacterium, *Bacillus anthracis* [1]. Spores can persist for long periods, even decades, under certain environmental conditions [2]. Anthrax occurs nearly worldwide, except Antarctica, including in: some Mediterranean countries; parts of Canada and the United States of America (USA); some countries of central and South America, central Asia, western China, and several sub-Saharan African countries [1,3]. Several eastern African countries have reported anthrax and estimated areas at risk [4,5,6]. In Kenya, anthrax is endemic with its burden felt in livestock production, public health, and wildlife conservation, resulting in its ranking as the highest priority zoonosis in the country [7]. Areas of endemic anthrax can be broadly characterized by seasonal rainfall and dry periods [8,9], with pathogen persistence associated with higher pH and more organic soil conditions and topography [1,2,10,11]. A recent ecological niche modelling study estimated areas with environmental conditions suitable for anthrax outbreaks cover 22% of Kenya’s landmass [12]. Livestock and wildlife outbreaks are frequent with varying intensity and host composition throughout Southern Kenya [13,14]. However, an investigation on the impacts of climate change on Kenya’s future anthrax risk is missing.

The global climate is undergoing unprecedented changes with shifting precipitation patterns [15], and an expected mean temperature increase of 1.5 °C by the end of the year 2100 [16]. Climatic changes are projected to lead to a global rise in the prevalence and the potential for geographic range expansions of infectious diseases [17]. Range contractions are also predicted where future conditions may no longer support host, vector, or pathogen ranges [18,19]. Within these changing ranges (expansion or contraction), changing climatic conditions are expected to affect the intensity and location of infectious disease outbreaks as well as diffusion range, amplification, and persistence in new habitats [20,21,22]. Recently, due to climate change, permafrost in the Arctic has melted, exposing hitherto preserved *B. anthracis* spores with anthrax outbreak consequences [23]. Shifts in seasonal extremes (hot, cold; dry, wet) potentially stimulate pathogen metabolic capacity [24]. Extreme weather patterns also significantly impact soil composition and vegetation [25], which can influence animal grazing behavior resulting in possible ingestion of *B. anthracis* spore-laden soils.

Effects of climate change and variability on infectious diseases have recently attracted substantial interest, particularly on the African continent [17,20,26]. Studies on impacts of disease transmission due to increase in temperature and rainfall in East Africa have been undertaken in which an epidemic of Rift Valley Fever (RVF) in Kenya and reduced malaria transmission in the United Republic of Tanzania were confirmed [27,28]. While there are extensive studies on the future spatial patterns of vector-borne infectious diseases due to climate change [29], fewer have been undertaken for environmentally mediated bacterial diseases, such as anthrax, where infectious *B. anthracis* spores can persist long term. The few studies that have investigated climate change impacts on anthrax outbreaks focused their efforts on the USA, Kazakhstan, Bosnia-Herzegovina, and the Northern Hemisphere [22,30,31,32]. There is evidence that Kenya has experienced climate change in the recent past due to rising temperatures, changing rainfall patterns, and increasing frequency of droughts and flooding [33].

Understanding how spatial patterns of pathogens, host populations, or vectors may change with a changing climate is important for disease control planning. Statistical models for mapping species’ distributions, such as ecological niche models (ENMs) are frequently applied for this purpose. Broadly, ENMs are modeling approaches aimed at predicting a species’ potential geographic distribution on a selected landscape by pattern matching or statistically correlating species’ presence locations to environmental variables to determine suitable environmental conditions that meet the species’ ecological requirements [30,34]. Those requirements are then mapped onto the landscape to predict the areas of relative habitat suitability [35]. Simply stated, species’ variable space relationships projected onto the landscape provides a testable hypothesis in biogeography [36]. Numerous ENM machine learning and rule-based algorithms, such as boosted regression trees (BRT) and genetic algorithm for rule-set production (GARP), have been developed for and implemented in predicting various taxa’s potential geographic distributions utilizing presence-only data and environmental covariates. These ENM techniques and others are increasingly used in the spatial epidemiology field, particularly for *B. anthracis* [37,38]. *Bacillus anthracis* has been predicted globally and across several countries: Australia, USA and Mexico, China, Ghana, Italy, Kazakhstan, Kyrgyzstan, West Africa, Tanzania, and Zimbabwe [11,22,37,39,40,41,42,43,44,45]. Specifically, BRT modeling has also been previously applied in predicting infectious diseases’ distributions under climate change scenarios [46,47]. A study [12], utilized BRT to predict the current day distribution of anthrax on southern Kenya’s landscape showing higher probabilities of occurrence between the western highlands and the shared Kenya-Tanzania border. Expanding their study to consider future climates is imperative to identify potential expansions or contractions of the geographic distribution of anthrax outbreaks in Kenya.

Future global climate models (GCMs), developed under different scenarios of how temperature and greenhouse gases will change, provide environmental covariates to examine how geographic distributions of a species (e.g., *B. anthracis*) may change over time [48]. Following the Intergovernmental Panel on Climate Change (IPCC) fifth assessment report (AR5), future climatic conditions are estimated through radiative forcing climate scenarios that include four different representative concentration pathways (RCPs) of greenhouse gas emissions (GHG) and atmospheric concentrations in the year 2100. These RCPs vary how the climate will change, from the most stringent societal mitigation efforts to the least (RCP 2.6 and RCP 8.5, respectively), with two intermediate RCPs (4.5 and 6) [49].

This study’s objective was to determine the geographic distribution of anthrax in Kenya under current and future mitigated and unmitigated climatic scenarios based on the regional circulation model climate predictions downscaled for Africa for year 2055. Such ENM-based predictions can be used to anticipate mitigation measures to reduce future anthrax outbreaks in Kenya.

## 2. Materials and Methods

### 2.1. Study Area

**Figure 1:**
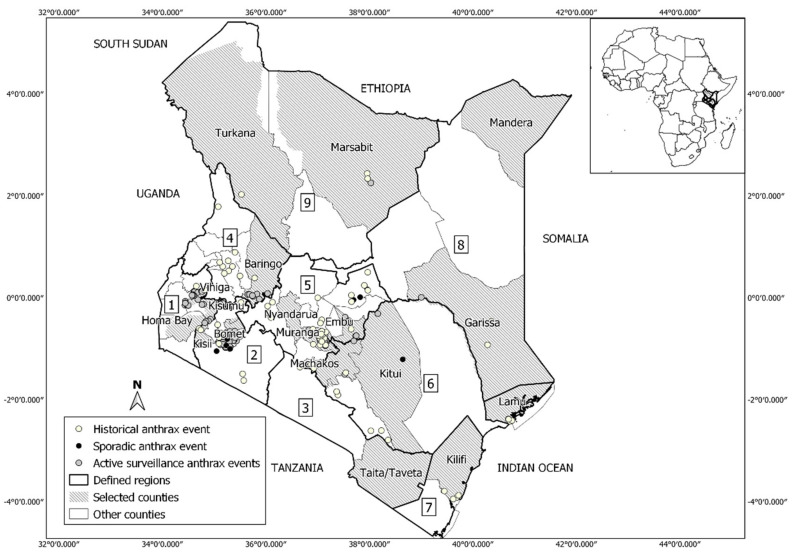
Map of Kenya showing study counties and the spatial distribution of anthrax occurrence data: historical (yellow dots) recorded between 2011 and 2017, sporadic surveys (black dots) recorded between 2018 and 2019, active surveillance (grey dots) recorded between 2019 and 2020. The 18 selected counties represent randomly selected counties stratified on agroecological zones to undertake anthrax outbreak active surveillance. Defined regions 1–9 arbitrarily represent important regions for describing the predicted distribution of anthrax: (1) Lake Victoria basin; (2) Southwestern; (3) Southern; (4) Western; (5) Central; (6) Eastern; (7) Coastal; (8) Northeastern; (9) Northern.

Our study includes all of Kenya with an area of ~580,367 km^2^. Kenya lies on latitude and longitude 1°00′ N and 38°00′ E. Figure 1 shows administrative units of Kenya and anthrax outbreak locations used in this study.

Kenyan climatic conditions vary from humid tropical at the coastal areas through temperate and sub-tropical inlands to hot and dry in arid and semi-arid regions. Kenya experiences a long rainy season from March to June and a short rainy season from September to December. In recent years, there have been variations in seasons due to climate change or shift with precipitation maintained at the same intensity but with varied distribution in space and time. Temperature has increased in variability with an estimated increase of 1.0 °C since 1960 representing average rate of 0.21 °C per decade [50].

### 2.2. Anthrax Occurrence Data

Georeferenced anthrax occurrences (presence) data were obtained from historical archives (n = 86), sporadic outbreak surveys (n = 13), and active surveillance (n = 119). Historical data were obtained from the Kenyan Directorate of Veterinary Services (DVS) covering 2011 to 2017, sporadic outbreaks data were collected through field surveys at outbreak sites between 2017 to 2018 and active surveillance data were collected through regular mobile phone transmissions of outbreaks from all wards of the 18 randomly selected counties between 2019 and 2020. Presence points were spatially thinned to ensure that each predictor pixel contained only one point (n = 178), then an equal number of pseudo-absence points were randomly generated at least 5 km (Euclidian distance) away from these presences. Outbreak data were initially recorded in Microsoft Excel and converted CSV files for use in modelling experiments. Pseudo-absence points were drawn at the beginning of each run within the modelling process and combined with presence points ahead of model development. Presence points and subsequent model outputs were mapped in QGIS version 3.1.6.0 [51].

### 2.3. Predictive Data and Variable Selection

A total of 69 publicly available bioclimatic variables for current and future projections were downloaded from https://webfiles.york.ac.uk/KITE/AfriClim/GeoTIFF_30s/ (accessed on 5 December 2020) (1 km resolution at the equator) [16], along with elevation derived from a digital elevation model (DEM) from https://doi.org/10.5065/A1Z4-EE71 at 1 km (accessed on 5 December 2020) [52], (Table S1). The bioclimatic data provide biologically significant variables encompassing seasonality, annual ranges, and limiting factors applicable for niche modeling, including monthly and annual variables for temperature, precipitation, and extremes [53]. The data comprised current (1961–1990) and future scenarios of Mid-century (2041–2070), africlim_ensemble_v3_[base]. The mean ensemble spans over ten GCMs, downscaled with five regional climate models and four contemporary currents to effectively reduce biases [52]. The future scenarios, RCP 4.5, and RCP8.5 were selected to compare an intervention and a non-intervention scenario of GHG emissions. The RCP 4.5 scenario represents reduced GHG emissions by interventions through the employment of a range of technologies and strategies leading to stabilization without overshoot pathway to 4.5 W/m^2^ in 2100, while RCP 8.5 scenario represents no intervention resulting in a worst-case of high GHG emissions of rising radiative forcing pathway leading to 8.5 W/m^2^ in 2100 [49]. Elevation was selected as an environmental variable that would remain constant into the future and was used to derive slope. All the data were then subset to the study area.

We used the variance inflation factor (VIF) to test candidate predictor variables for multicollinearity at the cut-off of VIF < 10 and to reduce highly correlated variables from the current data. A VIF is produced by regressing variables against each other and increasing VIF values above one indicates coefficient variance higher than would be expected with zero collinearity [53]. Cut-off values for multicollinearity in VIF are debated, and values above ten are considered highly correlated [54,55]. Due to the bioclimatic variables’ derivation process, some collinearity was expected, and using the standard VIF cut-off deemed appropriate. Values from all final environmental covariates were extracted to presence and pseudo-absence points using the ‘raster’ package in R ahead of the model building. Variable preparation, analysis, and modelling was performed R version 4.0.3 [56] and QGIS version 3.1.6.0 [51].

### 2.4. Model Building and Evaluation

Here we used boosted regression trees (BRT) to estimate the current distribution of anthrax in Kenya and subsequently project changes in 2055 under each RCP 4.5 and RCP 8.5. Briefly, BRTs implement regression trees and boosting to progressively assemble and consolidate many simple decision trees [57]. Thus, BRTs utilize statistical and machine learning methods to refine prediction estimates by coalescing large numbers of shallow trees. The performance of a BRT experiment can be further enhanced by tuning several hyperparameters (values used to control the model learning process) detailed in [58]: bagging fraction (bf) introduces randomness into the model by defining the proportion of data drawn at random from the original data at each step, thereby improving performance and reducing overfitting; tree complexity (tr) specifies the number of nodes for each tree; learning rate (lr) varies the contribution of each tree added to the model. A lower learning rate resulting in a higher number of trees is preferable when several observations and computational time are available for model fitting.

We built BRTs using the ‘gbm’ package (‘gbm.step’ extension) in R [59]. We employed bootstrapping, also called an ensemble approach, to generate and evaluate 100 individual BRT experiments and average the result for a best spatial prediction of the distribution of anthrax under current and future conditions. For each experiment, new pseudo-absence data were randomly generated and combined with the presence data. The combined data were then partitioned into model training (75% of the data) and model evaluation testing sets (25% of the data).

We assessed ‘gbm.step’ function settings for bagging fractions, learning rate, and tree complexity in exploratory experiments using minimum predictive error to obtain the best predictive performance based on training AUC. The final ‘gbm.step’ was thus set to fit the training data with learning rate (lr) = 0.001, bagging fraction (br) = 5 and maximum tree = 2500. Model performance was evaluated using AUC (area under the curve) ROC (receiver operating characteristics) curves for each experiment and averaged across all experiments. AUC is considered the most prominent among ENM prediction evaluation methods, despite its identified drawbacks such as assigning equal weights to omission and commission errors [60,61]. The predictions for model experiments (n = 100) were generated and averaged to obtain the final anthrax distribution map for the study area; the lower 2.5% and upper 97.5% confidence intervals were also mapped.

Partial dependency plots (PDPs) were generated to graphically illustrate the functional relationship between the target response (presence of anthrax) and the set of predictors [62]. All PDPs were generated with the ‘pdp’ R package [62] for each individual and across experiments to demonstrate how each predictor influenced mean prediction probabilities and the strength of its contribution to the prediction.

Anthrax distribution in Kenya was mapped for models fitted with the selected current climatic scenarios and slope as predictor variables. Future scenarios were mapped to the landscape by substituting the current climatic variables with the corresponding RCP 4.5 and RCP 8.5 climatic variables based on the current BRT models. Final predictions were dichotomized as high risk (or likelihood of supporting anthrax) for any pixel exceeding the Youden index derived threshold [63]. We examined potential spatial shifts in anthrax risk areas from current to present in two ways. First, we calculated the standard deviational ellipses for all pixels identified as high risk for the current and each future prediction. Next, we overlaid the current and both future predictions and color-coded pixels as stable (present in the current and both future distributions), loss (present in current lost in either one or both future predictions), or expansion (anthrax risk absent in the current prediction and present in one or both future predictions). To visualize the overall direction of expansion under current and future climatic conditions, standard deviational ellipses were calculated in R using the ‘aspace’ package [64] and mapped over the BRT predictions.

## 3. Results

### 3.1. Model Variables

The VIF analysis filtered the 71 candidate variables to 10 independent variables (Table 1), which were finally fitted in the BRT experiments.

### 3.2. Prediction of Potential Anthrax Distribution Due to Climate Changes

**Figure 2:**
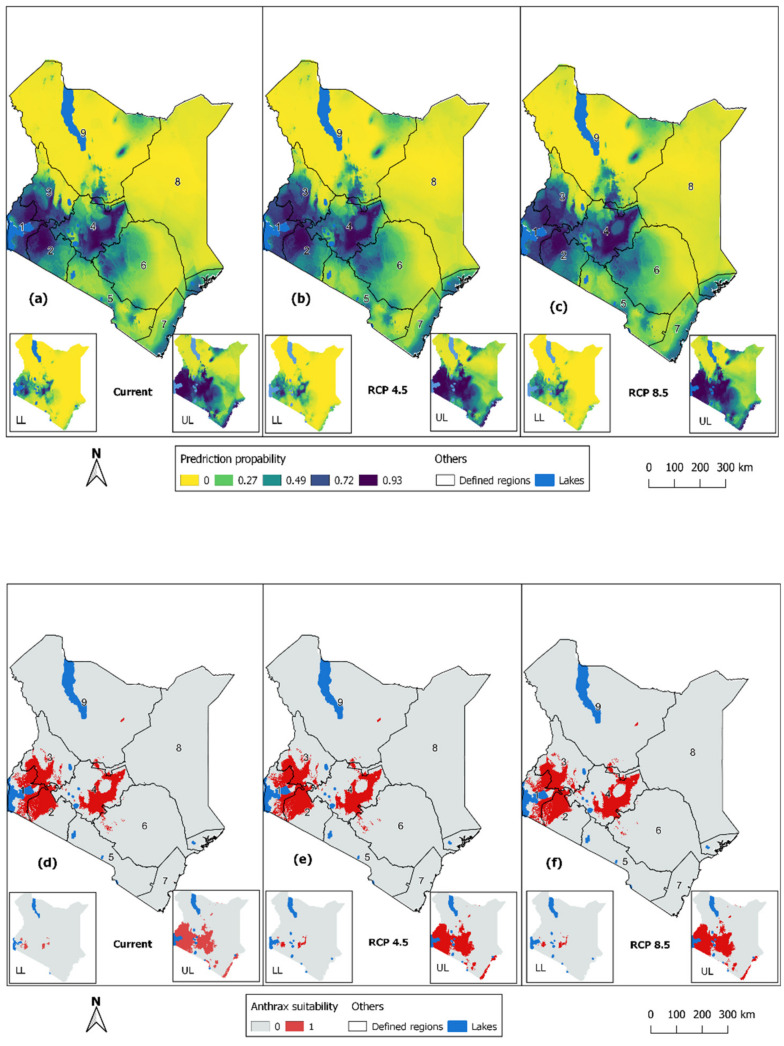
Potential distribution predictions for anthrax occurrence in Kenya for climate scenarios: (**a**) the current climate; (**b**) future RCP 4.5; (**c**) Dichotomized predictions of anthrax suitability using the Youden index (≥0.75) for climate scenarios: (**d**) the current climate; (**e**) future RCP 4.5; (**f**) RCP 8.5. Inset maps for each panel show the lower (2.5%; **left**) and upper (97.5%; **right**) confidence intervals of predictions. Codes 1–9 indicate arbitrarily defined regions to represent important regions for describing the predicted distribution of anthrax: (1) Lake Victoria basin; (2) Southwestern; (3) Southern; (4) Western; (5) Central; (6) Eastern; (7) Coastal; (8) Northeastern; (9) Northern.

The mean predicted anthrax distribution from the current scenario had a mean training AUC of 0.936 ± 0.0019 and a mean test AUC of 0.929 ± 0.0039. The subsequent predictions for future scenarios were projected onto the landscape based on the current BRT models and future climatic covariates. As illustrated in Figure 2a–c, the areas predicted to be highly suitable for anthrax at prediction probability ≥0.75 (Youden index) for the three scenarios, were predominantly in central regions bordering the central highlands of Kenya; western regions around Lake Victoria, and the western highlands bordering Uganda; and the southwestern region along the Kenya-Tanzanian border. Lower suitability at probability <0.75 was predicted for the eastern region further from central Kenya, the southern region bordering Tanzania, the coastal region away from Indian ocean, and the northern and north-eastern region. Figure 2d–f show high and non-risk areas dichotomized at the Youden index threshold (≥0.75).

### 3.3. Variable Contribution

**Figure 3:**
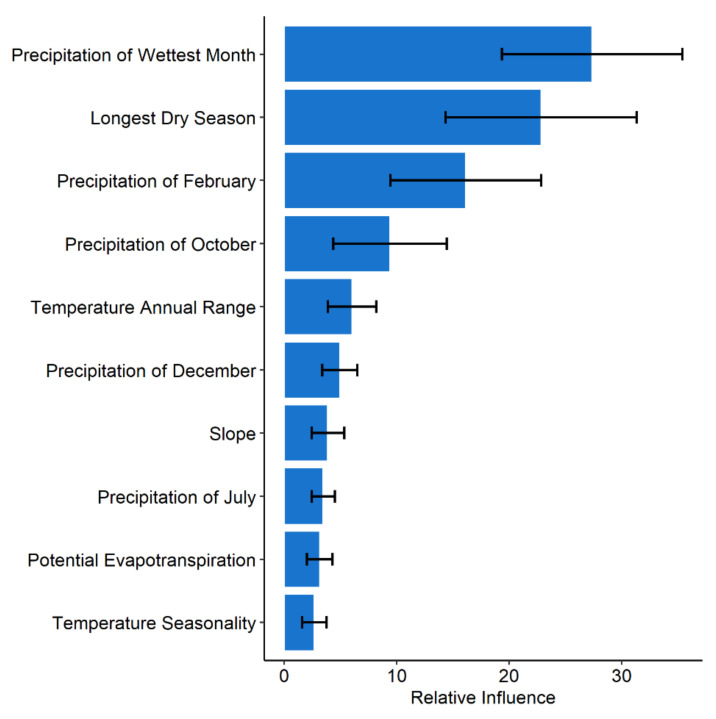
Variable relative influence for final variable set used to model the distribution of anthrax in Kenya using boosted regression tree experiments. Error bars represent variability across an ensemble of 100 BRT experiments.

Relative variable influence across the 100 experiments is illustrated in Figure 3 in order of their importance. Precipitation of wettest month is identified as the top ranked and temperature seasonality the least important among the 10 variables across the experiments.

### 3.4. Marginal Effect of the Climatic Variables on Anthrax Distribution Predictions

**Figure 4:**
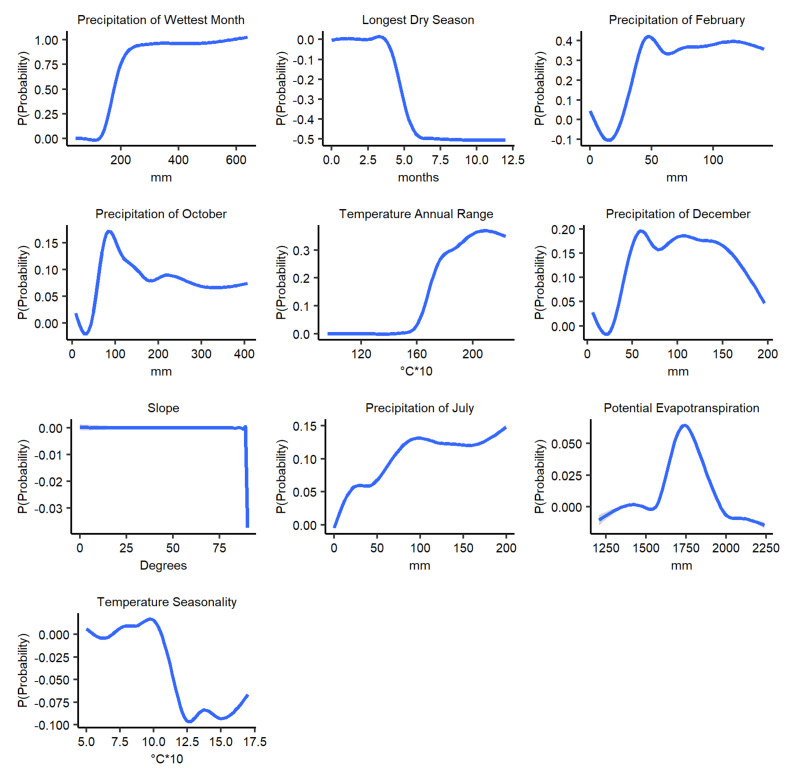
Partial dependency plots (PDP) showing marginal effects on the prediction probability of potential anthrax distribution by each variable across the 100 BRT experiments for current climatic conditions.

Partial dependency plots (PDP) showing the marginal effects of each variable on anthrax prediction probability, while keeping all other variables at their average, are illustrated in Figure 4. In general, the relationships between each of the variables and the prediction probability were nonlinear and multimodal. Increased precipitation of wettest month (between ~150–200 mm), precipitation of February (between ~20–50 mm), July (between ~0–100 mm), October (between ~20–100 mm), and December (between ~30–50 mm), annual temperature range (between ~16–28 °C), and potential evapotranspiration (between 1500 mm to 1750 mm) were associated with higher probability of anthrax suitability. On the other hand, increased longest dry season (between ~3–6 months) and temperature seasonality (between ~1–1.3 °C) were associated with decreasing anthrax prediction probability. Slope exhibits a constant relationship before a constant drop after 85 degrees with the prediction probability.

### 3.5. Change Detection

**Figure 5:**
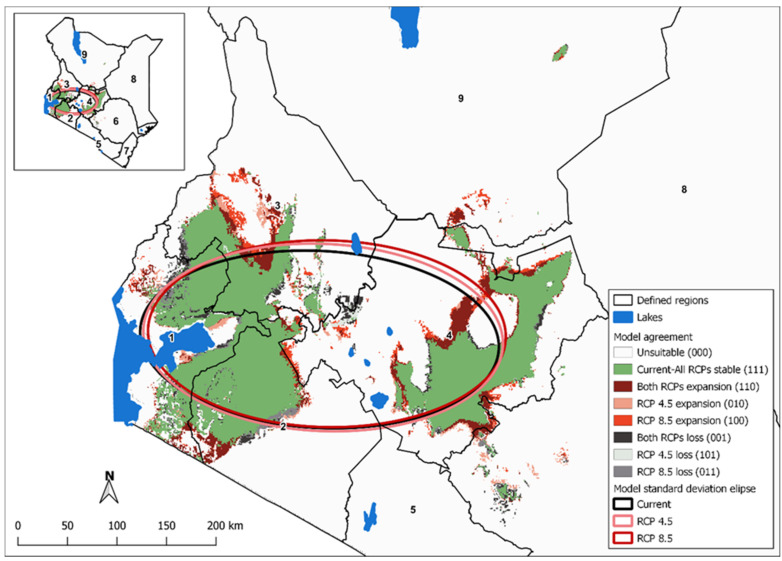
The model agreement between the current and future Youden dichotomized anthrax suitability predictions. Unique raster values summations represented agreement of current, RCP 4.5, and RCP 8.5 predictions. Standard deviational ellipses show directional distribution trends for the projections.

The change detection between the current and the two future climatic scenarios showed some varied differences in the potential distribution of anthrax risk in the future. The predicted high-risk areas at probability ≥0.75 for current, RCP 4.5, and RCP 8.5 scenarios were 36,131 km^2^, 40,012 km^2^, and 39,835 km^2^, respectively. In Figure 5, the approximated areas out of the of study area (~580,367 km^2^) for the predicted anthrax distributions of current and both future scenarios, exhibited: 6% of no-change; 0.9% of expansions; and 0.4% of losses. Also, there was a northward shift from current to RCP 8.5 prediction.

## 4. Discussion

This study used boosted regression tree modelling to predict the potential spatial distribution of anthrax in Kenya based on current climate conditions (Baseline (1961–1990) and two future RCPs (4.5 and 8.5) for the year 2055. Based on the Youden index, areas of agreement for anthrax distributions under current and future climate change scenarios were ~6% of the study area (~580,367 km^2^). Areas, where there were expansions and losses, were also predicted at ~0.9% and ~0.4% of the study area, respectively. The predicted agreement areas also covered areas that had been reported with anthrax outbreaks in previous studies in Kenya [13]. These overlapping report regions suggest that anthrax outbreaks tend to reoccur in the same localities, likely due to the persistence of spores in the soil [65]. A study suggested that anthrax spores exposed to the surface from flooding due to increased precipitation and spore concentration from dry season might have contributed to anthrax outbreaks in Bosnia–Herzegovina [31]. Our study also suggests precipitation and dry seasonality as key variables for predicting the extent of anthrax in Kenya. Like our research, a study on climatic influence on anthrax suitability in the Northern Hemisphere also predicted the expansion of suitable anthrax areas under future climatic scenarios [32]. Kenya and the Northern Hemisphere have environmental differences; however, there are commonalities in patterns of climate-sensitive infectious diseases in the Arctic and the tropics [66].

Our study suggests a possible northward shift in anthrax distribution between the current and the two 2055 climatic scenarios towards low arid and semi-arid areas (ASALs). An increase in risk was also suggested from both the future climatic scenarios in portions of the areas that already had high-risk predictions in the central region and western region bordering Uganda. Interestingly an increase in risk was suggested in the northern region, an area that did not already have high-risk predictions. On the other hand, a reduction in risk was shown for both future scenario predictions in small patches of western, central, coastal, and southwestern regions. Similar climate change prediction studies, though based on RVF, showed similar variation in disease risk suitability across Kenya and Tanzania [26,67]. It is worth noting that anthrax and RVF are both influenced by similar climatic factors of precipitation, temperature, and their derivatives [2,26,68]. The suggested emergence of risk in new areas in ASALs with respect to future climate change scenarios may be attributed to the effect of micro-climatic conditions influenced by the relatively elevated altitude of the neighboring rift valley escarpments. Climate changes have been confirmed to alter temperatures regimes, precipitation patterns, and other climate variables [16], which can in turn, define livestock-human interface areas, the meeting of infected hosts, and transmission season of anthrax with anthrax outbreaks increase potential [69]. In Kenya, changes in temperatures, rainfall patterns, frequency of droughts, and flooding have been recorded [33].

Our study suggests an increasing marginal effect of precipitation variables and annual temperature range with the probability of anthrax distribution prediction at specific ranges. On the other hand, a decreasing marginal effect for length of longest dry season and temperature seasonality. This may indicate that areas that receive large quantities of rain in a short period but have prolonged droughts may or may not support anthrax well due to the competing marginal effects. Temperature and rainfall trends, seasonality, and extremes have been found to determine anthrax outbreak distribution in previous studies [5,32,41,42]. An increase in precipitation has been found to influence anthrax outbreaks by exposing buried spores to the surface or increase run-offs that collect and concentrate spores in ‘storage areas’ and possibly disperse them [70], though evidence on the storage area hypothesis is limited.

Potential evapotranspiration is suggested in our study to have an increasing marginal effect with prediction probability peaking at 1750 mm before decreasing. This indicates that low potential evapotranspiration values below 1750 mm are important in defining anthrax distribution in Kenya. Potential evapotranspiration has been found to predict anthrax suitability in other environments [44,71]. Potential evapotranspiration defines the ideal evaporation realized with sufficient water availability reflecting interludes of precipitation and air temperature, influencing sporulation and multiplication of *B. anthracis*. Slope exhibited a constant marginal effect on anthrax’s prediction probability up to 80 degrees in our study, perhaps suggesting a uniform contribution with rainfall seasonality on anthrax distribution in Kenya. Slope defines terrain steepness or flatness and can control the flow of spore laden run-offs to shallow depressions in the local topography [72].

While our study has produced several important insights on the potential present and future spatial distribution of anthrax outbreaks in Kenya, there are several limitations. Like elsewhere, anthrax reporting may underestimate outbreaks in Kenya, requiring greater effort to improve reporting nationally. The pseudo-absences generated as absences might represent presence locations [73]. This limitation was minimized by locating the pseudo-absences at least 5 km (Euclidian distance) away from any presence point (known outbreak) assuming the distance would reduce overlap. Additionally, the pixel resolution of environmental covariates may limit detection of local anthrax areas important for disease control. Additionally, refinement of global climatic condition estimates is hampered by the poor distribution of synoptic meteorological stations that could provide primary data for improving such estimates. There are inherent uncertainties associated with general circulation models (GCMs) as each GCM relies on specific parameters and functions to project the climatic scenarios. We attempted to correct this by applying africlim_ensemble_v3_[base] that uses ten GCMs to reduce biases [53]. RCPs are also associated with some inherent area uncertainties [74].

This study generated potential anthrax distribution maps from current and future climatic conditions as a proxy for risk maps that can be integrated into policy frameworks for prospective targeted anthrax surveillance and control in identified risk areas. This approach might shift the paradigm of decision-making from a reactive, often triggered by prevailing health and safety challenges, to a proactive one, which is spatially informed and cost-effective. Maps of disease risk can be used to prioritize surveillance and control actions to only identified target areas considering limited resources. Measures that should be enhanced for better control of anthrax include regular annual vaccinations of livestock and community education programs that may reduce the suitability to future outbreaks, possibly mitigating some of the effects of climate change.

## 5. Conclusions

Our study predicted potential anthrax distribution areas as influenced by climate change in Kenya. These findings present risk maps that can be used to mitigate future anthrax outbreaks through anticipatory targeted surveillance and control to minimize the impact of anthrax in the region. Furthermore, surveillance should be intensified in these high-risk areas.

## Figures and Tables

**Table 1:** Variables fitted in BRT algorithm for niche modeling.

Variable	Unit
Precipitation of wettest month	mm
Temperature Seasonality	°C*10
Annual temperature range	°C*10
Length of longest dry season	months
Potential evapotranspiration	mm
Mean precipitation of October	mm
Mean precipitation of December	mm
Mean precipitation of February	mm
Mean precipitation of July	mm
Slope	degrees

## Data Availability

Environmental data presented in this study are openly available in Harvard Dataverse at https://doi.org/10.7910/DVN/7WOXRG (accessed on 4 March 2021). The anthrax occurrence data presented in this study are available on request from the corresponding author. The data are not publicly available due to requirement for authorization from Directorate of Veterinary Services. Kenya.

## Supplementary Materials

The following are available online. Table S1, showing initial 71 candidate variables their sources, references, and descriptions.
