# Supplementary material for: Modeling the Potential Future Distribution of Anthrax Outbreaks under Multiple Climate Change Scenarios for Kenya
Source: Int J Environ Res Public Health. 2021 Apr 15;18(8):4176. doi: 10.3390/ijerph18084176 (PMC8103515; doi:10.3390/ijerph18084176)
Supplement: Supplementary file 1 [file ijerph-18-04176-s001.pdf]

Scheme 1. Tables. Data description and online source.

	Variable	Source	Code	Unit
1.	30-arc seconds Mean annual temperature	1	BIO1	C° x10
2.	30-arc seconds Mean temp warmest quarter	1	BIO10	C° x10
3.	30-arc seconds Mean temp coolest quarter	1	BIO11	C° x10
4.	30-arc seconds Mean annual rainfall	1	BIO12	mm
5.	30-arc seconds Rainfall wettest month	1	BIO13	mm
6.	30-arc seconds Rainfall driest month	1	BIO14	mm
7.	30-arc seconds Rainfall seasonality	1	BIO15	mm
8.	30-arc seconds Rainfall wettest quarter	1	BIO16	mm
9.	30-arc seconds Rainfall driest quarter	1	BIO17	mm
10.	30-arc seconds Mean diurnal range in temp	1	BIO2	C° x10
11.	30-arc seconds Isothermality	1	BIO3	C° x10
12.	30-arc seconds Temperature Seasonality	1	BIO4	C° x10
13.	30-arc seconds Maximum temp warmest month	1	BIO5	C° x10
14.	30-arc seconds Minimum temp coolest month	1	BIO6	C° x10
15.	30-arc seconds Annual temperature range	1	BIO7	C° x10
16.	30-arc seconds Number of dry months	1	DM	months
17.	30-arc seconds Length of longest dry season	1	LLDS	months
18.	30-arc seconds Annual moisture index	1	MI	index x100
19.	30-arc seconds Moisture index arid quarter	1	MIAQ	index x100
20.	30-arc seconds Moisture index moist quarter	1	MIMQ	index x100
21.	30-arc seconds Potential evapotranspiration	1	PET	mm
22.	30-arc seconds Monthly precipitation	1	pr	mm
23.	30-arc seconds Monthly 2-metre air temperature	1	tas	C° x10
24.	30-arc seconds Monthly average of daily maximum temperature	1	tasmax	C° x10
25.	30-arc seconds Monthly average of daily minimum temperature	1	tasmin	C° x10
26.	30-arc seconds digital elevation model (GOTOPO30) (USGS)	2	DEM	m
27.	Slope (derived from GOTOPO30)	3	Slope	degrees

[1]https://webfiles.york.ac.uk/KITE/AfriClim/GeoTIFF_30s/;
[2]https://earthexplorer.usgs.gov/; [3]GIS Spatial analysis
